# Production of Sentential Negation in German and Italian Non-fluent Aphasia

**DOI:** 10.1007/s10936-022-09894-4

**Published:** 2022-06-25

**Authors:** Valantis Fyndanis, Gabriele Miceli, Rita Capasso, Helene Killmer, Sonia Malefaki, Kleanthes K. Grohmann

**Affiliations:** 1 Department of Rehabilitation Sciences, Cyprus University of Technology, Limassol, Cyprus; 2 MultiLing/Department of Linguistics and Scandinavian Studies, University of Oslo, Oslo, Norway; 3 Department of Linguistics, University of Potsdam, Potsdam, Germany; 4 Department of Psychology and Cognitive Science, University of Trento, Trento/Rovereto, Italy; 5 SCA Associates, Rome, Italy; 6 Department of Mechanical Engineering and Aeronautics, University of Patras, Rio Patras, Greece; 7 Department of English Studies, University of Cyprus, Nicosia, Cyprus

**Keywords:** Negation, Polarity, Non-fluent aphasia, Sentence anagram task, German, Italian, Verbal working memory, Education

## Abstract

The ability of persons with non-fluent aphasia (PWAs) to produce sentential negation has been investigated in several languages, but only in small samples. Accounts of (morpho)syntactic impairment in PWAs have emphasized various factors, such as whether the negative marker blocks or interferes with verb movement, the position of the Negation Phrase in the syntactic hierarchy or the interpretability of negation. This study investigates the ability of German- and Italian-speaking PWAs to construct negative sentences, as well as the role of verbal working memory (WM) capacity and education in task performance and production of sentential negation. German and Italian differ in the syntactic properties of the negative markers that are relevant here (*nicht* and *non*, respectively). A sentence anagram task tapping into the construction of negative and affirmative declarative sentences was administered to 9 German- and 7 Italian-speaking PWAs, and to 14 German- and 11 Italian-speaking age- and education-matched healthy volunteers. We fitted generalized linear mixed-effects models to the datasets. There was no significant difference between negative and affirmative sentences in either group of PWAs. There was a main effect of verbal WM capacity on task performance, but no interaction between verbal WM capacity and production of negative vs. affirmative sentences. Education did not affect task performance. The results are discussed in light of different linguistically-informed accounts of (morpho)syntactic impairment in non-fluent aphasia.

## Introduction

The ability of persons with aphasia (PWAs), especially those with non-fluent aphasia, to produce sentential negation has been investigated in several languages (e.g., Dutch: Bastiaanse et al., 2002; Rispens et al., 2001; English: Bastiaanse et al., 2002; Bebout, 1993; Jaichenco et al., 2020; Greek: Fyndanis et al., 2006; Koukoulioti, 2010; Stavrakaki & Kouvava, 2003; Norwegian: Bastiaanse et al., 2002; Rispens et al., 2001; Spanish: Bastiaanse et al., 2002; Jaichenco et al., 2020; Martìnez-Ferreiro, 2009; Catalan and Galician: Martìnez-Ferreiro, 2009; Japanese: Hagiwara, 1995; Italian: Chinellato, 2004), but most studies included very small samples of PWAs per language (n = 2–4) (e.g., Bastiaanse et al., 2002; Fyndanis et al., 2006; Hagiwara, 1995; Jaichenco et al., 2020; Koukoulioti, 2010; Rispens et al., 2001; Stavrakaki & Kouvava, 2003). Moreover, cross-linguistic differences in the accuracy performance of PWAs on tasks tapping into the production of sentential negation have been reported. For example, in Bastiaanse et al.’s (2002) study, English-speaking and Spanish-speaking PWAs were found to be significantly more impaired than Dutch-speaking and Norwegian-speaking PWAs on similar tasks tapping the construction of negative sentences. To account for these cross-linguistic differences, Bastiaanse et al. (2002) argued that PWAs (and especially persons with agrammatic aphasia) have difficulty producing negative sentences if the negative marker blocks verb movement or interferes with it. This is the case in English and Spanish, but not in Dutch and Norwegian. As explained in more detail in the section on *Syntax of sentential negation*, the negative marker blocks or interferes with verb movement when it occupies the head position of the Negation Phrase (NegP) in a given language, and the verb moves from a position below to a position above NegP, or is related to it.

Chinellato (2004) investigated the ability of five PWAs who spoke Standard Italian and an Italian dialect (Vicentino or Venetian) to produce preverbal and postverbal sentential negation in both Standard Italian and their Italian dialect. He found the PWAs to be impaired in preverbal sentential negation (which is relevant to the present study) in both language varieties. (Preverbal sentential negation is instantiated through the negative marker *non* in Standard Italian and through the negative marker *no* in the Italian dialects above.) Chinellato (2004) argued that this result is consistent with the theoretical view that the preverbal negative marker in Italian is a clitic (e.g., Belletti, 1990), which renders this negative marker prone to impairment in agrammatic aphasia. As will be described in the section *Syntax of sentential negation*, under Belletti’s (1990, 1994) analysis the Italian preverbal negative marker *non* is a clitic that is base-generated in the head position of NegP, adjoins to the verb and moves with it to a higher position. It thus interferes with verb movement. Therefore, under Belletti’s analysis, Chinellato’s (2004) results are consistent with Bastiaanse et al.’s (2002) account.

It should also be noted that studies investigating the ability of PWAs to produce sentential negation and focusing on the same language have produced contradictory results. For example, Fyndanis et al. (2006) found two Greek-speaking PWAs to be significantly impaired in the construction of sentential negation (in an indicative mood context), whereas Koukoulioti (2010) found three Greek-speaking PWAs to perform well on the production of negative sentences (in an indicative mood context). However, this discrepancy in Greek-speaking PWAs’ performance on sentential negation could reflect task effects. While Fyndanis et al. (2006) used sentence anagram tasks, Koukoulioti (2010) used a sentence elicitation task. Across-subject variability in agrammatic aphasia can also be observed in studies using the same method. For instance, Stavrakaki and Kouvava (2003) analyzed spontaneous speech samples of two Greek-speaking PWAs and found that one was 34.6% correct in contexts requiring sentential negation that is instantiated through the Greek negative marker *den* ‘not’, and the other was 72.2% correct in such contexts.

It is worth mentioning that, although some PWAs produce negative and affirmative sentences with comparable accuracy (see, for example, the Dutch-speaking and the Norwegian-speaking PWAs reported in Bastiaanse et al., 2002), this does not mean that producing negative and affirmative sentences is equally demanding. Psycholinguistic studies on negation have consistently found that negative sentences pose greater processing demands than affirmative sentences (e.g., Kaup & Dudschig, 2020, and references therein), which might be due to negative sentences being semantically and syntactically more complex than their affirmative counterparts (e.g., Xiang et al., 2020). However, the discrepancy between negative and affirmative sentences in processing demands does not always emerge in PWAs. This might be so because most studies on negation in aphasia selected accuracy performance as the dependent variable. Accuracy is not always sensitive to the fact that negation prolongs processing times (Kaup & Dudschig, 2020). Nevertheless, in previous studies on non-fluent aphasia, similar anagram tasks yielded dissociations between negative and affirmative sentences in some specific languages but not in others (see, for example, results reported in Bastiaanse et al., 2002, and Rispens et al., 2001). This suggests that language-specific syntactic factors related to sentential negation may pose additional demands on PWAs’ processing system, causing the relative difficulty of negative sentences to emerge even in less sensitive measures such as accuracy performance.

Negation is associated with longer processing times especially when negative sentences are processed out of context or when the context provided is not sufficiently supportive (e.g., Albu et al., 2021; Dudschig et al., 2021a; Kaup & Dudschig, 2020). The extent to which the provided context suffices to reduce the processing costs associated with negation is determined by the amount of informativeness in negative sentences given the context (e.g., Xiang et al., 2020) and the context-based expectations (e.g., Nordmeyer & Frank, 2014; Wason, 1965). It should be noted, however, that in Xiang et al.’s (2020) study on healthy participants, negative sentences were found harder to process than affirmative sentences regardless of their informativeness.

In the sentence anagram task with pictures used in Rispens et al. (2001) and Bastiaanse et al. (2002), which is relevant to the current study (see Materials and Methods section), the target negative and affirmative sentences, although not embedded in a verbal context, were accompanied by pictures, which provided nonverbal contexts. In other words, since the participant had to decide whether to construct an affirmative or a negative sentence based on the stimulus picture, the production of negative sentences was never out of context. In the negative polarity condition, participants always constructed negative sentences to negate a proposition that did not agree with the picture. Hence, in the negative polarity condition, participants were always provided with some “legitimizing contexts” for sentential negation. However, one could counter that, given the pictorial contexts provided in the task, negation was not “pragmatically licensed” in terms of informativeness of the target negative sentences (see Dudschig et al., 2021a) as the negative sentences never provided new information which could not be inferred from the picture, nor was it the most informative option to describe the state of affairs represented in the picture (Xiang et al., 2020). Moreover, pictorial contexts were minimal and the negative sentences only served to denote that the meaning representation of the picture did not match the negated proposition in the sentence. Therefore, in Rispens et al.’s (2001) and Bastiaanse et al.’s (2002) task, although negative sentences were tested within a context, probably such context was not supportive enough to minimize the processing demands posed by sentential negation. (For contexts that manage to do so, see Albu et al., 2021.) However, as can be seen in Bastiaanse et al. (2002), even if it is more challenging for PWAs to produce negative than affirmative sentences, not all PWAs show dissociations. This might be related to the insufficient sensitivity of accuracy as a dependent variable.

Besides the linguistic approaches to production of sentential negation, cognitive and/or demographic factors may also matter. For instance, verbal Working Memory (WM) can affect verb-related morphosyntactic production (e.g., Fyndanis, Arcara, et al., 2018; Kok et al., 2007), and cognitive inhibitory mechanisms seem to be involved in negation processing (Beltrán et al., 2018, 2019, 2021; Dudschig et al., 2021b). Furthermore, available evidence shows that education may affect performance on verbal tasks (e.g., Simos et al., 2011).

### The Present Study

This study adds to the body of literature on the production of sentential negation in non-fluent aphasia by investigating the ability of German-speaking PWAs and Italian-speaking PWAs to construct negative and affirmative sentences. A number of hypotheses have been put forward, which make different predictions for the patterns of impairment and preservation in the production of sentential negation in non-fluent aphasia. Before presenting these hypotheses and their predictions, some background information about the syntax of sentential negation is provided. Finally, since sentential negation may be taxing (Kaup & Dudschig, 2020), the current study also begins to address whether verbal WM capacity and education have significant main effects on accuracy performance on the production of negative vs. affirmative sentences, and whether either variable differentially affects production of the two sentence types.

### Syntax of Sentential Negation

The grammatical category associated with the distinction between affirmative and negative verb phrases or sentences is called *polarity*. All verb phrases or sentences have either an affirmative or a negative value of polarity. Affirmative is typically the unmarked polarity, whereas negative is the marked value. The grammatical rules that convert an affirmative sentence into a negative one vary across languages. In many languages, an affirmative sentence is converted into a negative one by adding a negative marker or particle meaning ‘not’. However, the ordering of this marker relative to the verb varies across languages. For instance, in German the negative marker *nicht*, which is used to form sentential negation, follows the verb (e.g., *Ich schlafe nicht* ‘I sleep-1st.sg. not (lit.)’ “I am not sleeping”), whereas in Italian the negative marker *non* precedes the verb (e.g., *Νon dormo* ‘Not sleep-1st.sg. (lit.)’ “I am not sleeping”). The difference in the relative ordering of the negative marker possibly reflects a structural difference between these languages. Negation is low in the syntactic tree of German, as NegP is directly above the Verb Phrase (VP) (Jäger, 2008), but high in the syntactic tree of Standard Italian (Zanuttini, 1997, 2001).

In Standard German, the negative marker *nicht* cannot block or interfere with verb movement, as it occupies the specifier position of NegP and-as mentioned earlier in this section-is located low (below the TP) in the syntactic hierarchy of German (Jäger, 2008; Jäger & Penka, 2012). As for Italian, contrastive views were presented. Zanuttini proposed that the preverbal negative marker *non* (e.g., *Anna non mangierà la pizza* ‘Anna not eat-FUT the pizza (lit.)’ “Anna will not eat pizza”), used to form sentential negation in Standard Italian, occupies the head position of NegP, which is located above the Tense Phrase (TP). On this view, the Standard Italian preverbal negative marker *non* does not block or interfere with verb movement in negative declarative sentences. A different analysis of sentential negation in Standard Italian has been proposed by Belletti (1990, 1994). Belletti (1990, 1994) agrees with Zanuttini (1997, 2001) that the preverbal negative marker *non* is base-generated in the head position of NegP in Standard Italian, but argues that NegP in Standard Italian is located below TP and that the preverbal negative marker *non* is a clitic (i.e. has an affixal status) that adjoins to the verb and moves with it to a higher position. Therefore, it does interfere with verb movement.

### Hypotheses and Predictions Regarding the Impairment vs Preservation of Sentential Negation in Non-fluent Aphasia

All hypotheses and predictions are summarized in Table 1. Bastiaanse et al. (2002) argued that PWAs have difficulty producing negative sentences if the negative marker blocks or otherwise interferes with verb movement. Since the German negative marker *nicht* does not interfere with verb movement (see “Syntax of Sentential Negation” section), Bastiaanse et al. (2002) would expect German-speaking PWAs to perform comparably well on the production of affirmative and negative sentences. Following Belletti’s (1990, 1994) analysis of sentential negation in Standard Italian, which posits that the negative marker *non* interferes with verb movement, Bastiaanse et al. (2002) would expect Italian-speaking PWAs to perform worse on negative sentences than on affirmative sentences. Based on Zanuttini’s (2001) analysis, however, Bastiaanse et al. (2002) would expect Italian-speaking PWAs to perform comparably well on the construction of negative and affirmative sentences.

**Table 1:** Summary of predictions connected to accounts of polarity/(morpho)syntactic impairment

	German-speaking PWAs	Italian-speaking PWAs
Bastiaanse et al. (2002)	negatives = affirmatives	negatives < affirmatives (under Belletti’s analysis) OR negatives = affirmatives (under Zanuttini’s analysis)
Fyndanis et al. (2012) (original IFIH)	negatives < affirmatives	negatives < affirmatives
Fyndanis, Arfani, et al. (2018) (revised IFIH)	negatives = affirmatives	negatives < affirmatives (under Belletti’s analysis) OR negatives = affirmatives (under Zanuttini’s analysis)
Friedmann and Grodzinsky (1997) (TPH), Hagiwara (1995)	negatives = affirmatives	negatives = affirmatives (under Belletti’s analysis) OR negatives < affirmatives (under Zanuttini’s analysis)

Under the Interpretable Features’ Impairment Hypothesis (IFIH) (e.g., Fyndanis et al., 2012; Nanousi et al., 2006; Varlokosta et al., 2006), both German-speaking and Italian-speaking PWAs should be impaired in the production of sentential negation as (1) polarity is interpretable and negation is the marked value of polarity, and (2) the IFIH (at least in the version proposed by Fyndanis et al., 2012) states that all morphosyntactic categories bearing interpretable features are demanding in terms of processing resources because they require processing and integration of both grammatical and extra-linguistic information. Thus, given that PWAs have reduced processing resources (e.g., Caplan & Hildebrandt, 1988; Fyndanis, Arcara, et al., 2018; Kok et al., 2007), the IFIH would expect both language groups of PWAs to produce sentential negation poorly.

However, under the revised version of the IFIH (Fyndanis, Arfani, et al., 2018), morphosyntactic categories bearing interpretable features are expected to be impaired in agrammatic aphasia only if they are instantiated through bound morphemes. According to Belletti’s (1990, 1994) analysis, the Italian negative marker *non* has an affixal status. Thus, it is considered to be a clitic and, therefore, it is a bound morpheme. Hence, under both versions of the IFIH (Fyndanis Varlokosta, & Tsapkini, 2012; Fyndanis, Arfani, et al., 2018) and following Belletti’s analysis, Italian-speaking PWAs should be impaired in sentential negation. In contrast, based on Zanuttini’s (2001) analysis, according to which the Italian negative marker *non* is a free-standing morpheme without affixal status, the revised version of the IFIH (Fyndanis, Arfani, et al., 2018) (but not the original IFIH; Fyndanis et al., 2012) would predict that Italian-speaking PWAs should perform similarly on negative and affirmative sentences. Finally, the German negative marker *nicht* is a free-standing morpheme without affixal status. Hence, only the original IFIH (Fyndanis et al., 2012) would expect German-speaking PWAs to be impaired in sentential negation.

Lastly, according to the *Tree Pruning Hypothesis* (TPH, Friedmann & Grodzinsky, 1997) and Hagiwara’s (1995) account, German-speaking PWAs are expected to perform comparably well on negative and affirmative sentences, as NegP is located low in the German syntactic hierarchy (Jäger, 2008). The TPH posits that agrammatic aphasia usually results from a pruning of the syntactic tree at the TP. While all nodes below the pruning site are preserved, tense and all nodes above TP become inaccessible. In a similar vein, Hagiwara (1995) argued that the higher in the syntactic tree a functional category, the costlier it is computationally and therefore the more prone to impairment it is. This is so because the higher in the syntactic tree a functional category is, the more the times that the operation *Merge* has to be implemented. Similarly, following Belletti’s (1990, 1994) analysis for Standard Italian, according to which NegP is located below TP, the TPH (Friedmann & Grodzinsky, 1997) and Hagiwara (1995) would expect Italian-speaking PWAs to perform comparably well on negative and affirmative sentences. In contrast, on Zanuttini’s (2001) analysis, according to which the NegP is high in the Italian syntactic hierarchy (in particular, above the TP), the TPH (Friedmann & Grodzinsky, 1997) and Hagiwara (1995) would expect Italian-speaking PWAs to perform significantly worse on negative than on affirmative sentences.

It should be noted that these predictions focus on PWAs, as in both languages, healthy participants should perform comparably well on negative and affirmative sentences despite the fact that negation is cognitively more demanding (e.g., Albu et al., 2021; Kaup & Dudschig, 2020, and references therein). Since neurologically unimpaired individuals have normal processing resources, and since accuracy performance is the outcome measure of our sentence anagram task, scores on negative sentences should not be influenced by negation-which could be the case if reactions times were considered. This prediction is in line with ceiling performance on a similar anagram task of the control groups reported in Rispens et al. (2001), Bastiaanse et al. (2002), and Fyndanis et al. (2006).

Finally, given that verbal WM affects verb-related morphosyntactic production (Fyndanis, Arcara, et al., 2018; Kok et al., 2007), and negation is cognitively demanding (Albu et al., 2021; Kaup & Dudschig, 2020), one would expect verbal WM to affect task performance by interacting with the production of negative and affirmative sentences. In particular, one would expect verbal WM capacity to affect negative sentences more than affirmative sentences. Moreover, since education has been found to affect performance on verbal tasks (e.g., Simos et al., 2011), a significant main effect of education on accuracy performance is also expected. Of relevance, education might be taken as a proxy for a procedural memory system that subserves language processing, namely *long-term WM for language* (Caplan & Waters, 2013). Building on Ericsson and Kintsch (1995), who coined the term *long-term WM* to refer to a memory system based on long-term memory storage and connected to skilled activities, Caplan and colleagues (see Caplan & Waters, 2013, and references therein) proposed that language is a skilled activity, and on-line syntactic processing is predominantly supported by long-term WM for language. They also suggested that WM, which is a controlled system, is recruited only at points of excessive processing demands. Although these ideas were developed to capture language comprehension data, it cannot be ruled out that long-term WM for language is also involved in aspects of language production, such as verb-related (morpho)syntactic production. For example, both controlled and procedural memory systems might subserve production of negative and affirmative sentences.

## Materials and Methods

### Participants

Overall, 16 PWAs and 25 healthy controls took part in the study. There were nine German-speaking PWAs and seven Italian-speaking PWAs, as well as 14 German-speaking and 11 Italian-speaking healthy volunteers. Each PWA language group was matched with the corresponding group of healthy controls on age and education. The study was approved by the ethics committee of the University of Potsdam. Participants gave informed consent in accordance with the Declaration of Helsinki. Demographic and (semi)spontaneous speech data for the German- and Italian-speaking participants are summarized in Tables 2 and 3, respectively. Presence and type of aphasia for the German-speaking PWAs were diagnozed based on clinical presentation and the results on the Aachen Aphasia Test (AAT; Huber et al., 1983, 1984). The aphasia diagnosis of the Italian PWAs was based on clinical presentation and the Italian version of the AAT (Luzzatti et al., 1996) and on the Batteria per l’Analisi dei Deficit Afasici (BADA; Miceli et al., 2006). Specifically, four Italian-speaking PWAs (P1, P2, P5, P6) were administered the AAT only, and three (P3, P4, P7) the BADA only. All German and Italian PWAs suffered from non-fluent (Broca’s) aphasia. Based on analyses of (semi)spontaneous speech-elicited through the Cookie Theft and the Stroke.

**Table 2:** German-speaking PWAs’ and control participants’ demographic and selected (semi)spontaneous speech data

	P1	P2	P3	P4	P5	P6	P7	P8	P9	Aphasic group (Mean (SD))	Control group (N = 14) (Mean (SD))
Demographic & cognitive variables											
Gender	F	M	F	F	M	F	F	M	F	6 F	9 F
Age (years)	43	75	38	50	70	60	63	62	47	56.4 (12.6)	62.1 (8.2)
Education (years)	11	8	11	11	11	11	17	17	17	12.7 (3.4)	13.6 (2)
Handedness	R	R	R	R	R	R	R	R	R	All R	All R
Etiology	Left ischaemic CVA	Left ischemic CVA	Left Ischemic CVA	Left ischemic CVA	Left ischemic CVA	Left haemorrhagic CVA	Left ischemic CVA	Right ischemic CVA	Left ischemic CVA	n.a	n.a
Aphasia post-onset (months)	194	227	68	255	32	59	5	258	40	126.4 (104.7)	n.a
Other conditions	Right hemiparesis	Right hemiparesis	No	Right hemiparesis	Right hemiparesis	Right hemiparesis	No	Left hemiparesis		n.a	n.a
Hearing/Vision	Normal	Normal	Normal	Corrected to normal	Normal	Normal	Normal	Normal	Normal	Normal or corrected to normal	Normal or corrected to normal
Diagnosis	Broca’s aphasia	Broca’s aphasia	Broca’s aphasia	Broca’s aphasia	Broca’s aphasia	Broca’s aphasia	Broca’s aphasia	Broca’s aphasia	Broca’s aphasia	n.a	n.a
Lesion site	Not available	Not available	Not available	Not available	Not available	Not available	Not available	Right fronto-parietal	Frontal–temporal & parietal	n.a	n.a
Verbal WM span	3	2	1	1	2	1	2	2	3	1.9 (0.8)	4.6 (1.2)
Language variables											
Words per minute	47.1	9.8	46.1	17.6	27.1	14.2	60	41.4	7.9	30.1(19)	117.7(22.3)
MLU	9.7	4.2	10.8	8.7	6.6	9.2	12.9	6.7	3.4	8 (3.1)	10.3(1.5)
%Grammatical sentences	27	0	3	20	13	40	20	10.8	12.5	16.3(12.3)	92(5.4)

*WM* working memory

**Table 3:** Italian-speaking PWAs’ and control participants’ demographic and selected (semi)spontaneous speech data

	P1	P2	P3	P4	P5	P6	P7	Aphasic group (Mean (SD))	Control group (N = 11) (Mean (SD))
Demographic & cognitive variables									
Gender	F	M	F	M	M	M	F	3F	5F
Age (years)	55	53	45	77	70	63	43	58 (12.6)	65.5 (10.6)
Education (years)	13	13	13	16	14	18	13	14.3 (2.0)	14.8 (4.2)
Handedness	R	R	R	R	R	R	R	All R	All R
Etiology	Hemorrhagic CVA	Left Ischemic CVA	Left Ischemic CVA	Left ischemic CVA	Left ischemic CVA	Left ischemic CVA	Left Ischemic CVA	n.a	n.a
Aphasia post-onset (months)	43	101	113	30	286	20	156	107 (93.2)	n.a
Other conditions	Right hemiparesis	Right hemiparesis	Right hemiplegia	Very mild right hemiparesis	Mild right hemiparesis	No	Right hemiplegia	n.a	No
Hearing/Vision	Normal/Corrected to normal	Normal	Normal/Corrected to normal	Normal/Corrected to normal	Normal/Corrected to normal	Normal	Normal	Normal/ Corrected to normal	Normal/ Corrected to normal
Diagnosis	Broca’s aphasia	Broca’s aphasia	Broca’s aphasia	Broca’s aphasia	Broca’s aphasia	Broca’s aphasia	Broca’s aphasia	n.a	n.a
Lesion site	Cortico-subcortical; fronto-parietal-Putamen	Cortico-subcortical; fronto-temporo-parietal-insula & Basal ganglia	CVA in left superficial & deep territory of MCA (middle & inferior frontal gyri, insula, superficial & lateral aspect of temporal pole, part of superior & middle temporal gyrus & white matter of above structures)	Left fronto-temporo-parietal	Left frontal involving Broca’s area & immediate surroundings	Left Frontal & Parietal	Left Frontal & Insular with extension to P	n.a	n.a
Verbal WM span	3	1	2	1	2	6	3	2.6 (1.7)	4.9 (0.7)
*Language variables*									
Words per minute	86.2	40.9	22.7	14.7	24.5	15.3	25.2	32.8(25.1)	93.6(28.5)
MLU	10	4.3	5.1	5.6	10.2	6.3	5.9	7.7(2.7)	16.7(4.6)
%Grammatical sentences	61.7	20.9	39.6	10.5	34.2	12.5	76	44(24.1)	91.9 (8.5)

*WM* working memory

Story-all PWAs had an agrammatic output, as indicated by their lower-than-normal proportion of grammatical sentences (see Tables 2, 3). Mean length of utterance was also below the normal range in 13 out of 16 PWAs. According to Faroqi-Shah and Thompson (2004), the combination of a relatively low proportion of grammatical sentences and a reduced mean length of utterance is consistent with a diagnosis of agrammatism. Spontaneous speech was elicited from control participants by asking them to describe the Cookie Theft picture and to narrate an important event of their life, either a pleasant or an unpleasant one. The analysis of (semi)spontaneous speech was based on syntactic, semantic and prosodic criteria, following the coding system developed by Thompson et al. (1995).

Verbal WM capacity was measured in all participants via a backward digit span task, which consisted of seven difficulty levels. German-speaking participants were administered the backward digit span task included in Härting et al. (2000). Italian-speaking participants were administered an Italian version of the backward digit span task that was administered to Fyndanis et al.’s (2018b) Italian-speaking participants.

### Task and Procedure

**Fig. 1:**
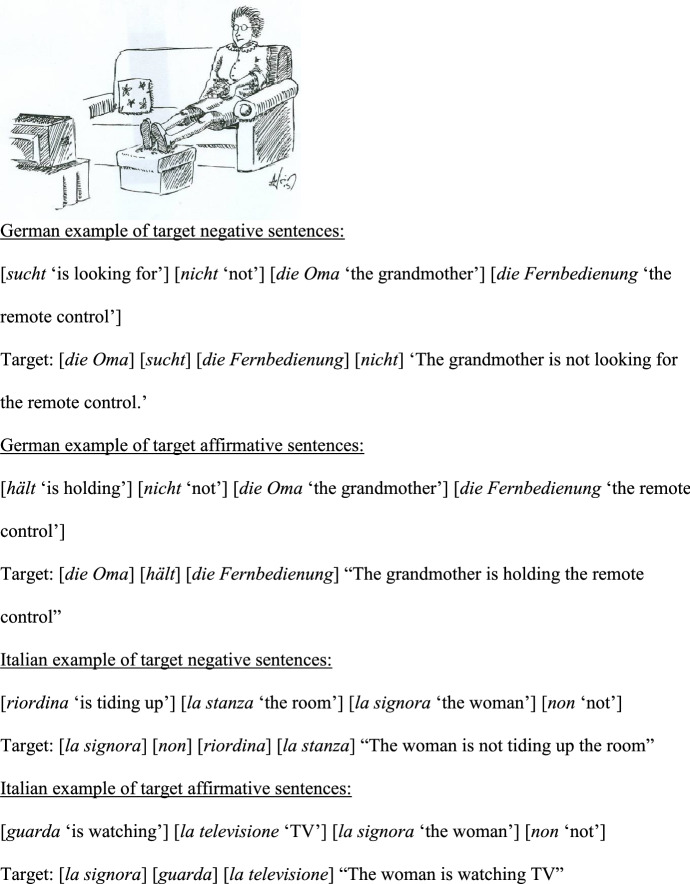
Examples of negative and affirmative sentences included in the sentence anagram task

Participants were administered a sentence anagram task (modelled after Bastiaanse et al., 2002, and Rispens et al., 2001) that required the construction of negative and affirmative declarative sentences. The German and Italian versions of the task consisted of 52 and 50 items, respectively. There were equal numbers of negative and affirmative sentences in each language version. Participants were presented with a picture and a set of cards (presented always in an non-grammatical order) that always contained the negative marker (*nicht* for the German version; *non* for the Italian version). They were asked to put cards in the correct order to construct a sentence and to decide, based on the picture, whether to use the negative element. (The task administration instructions-translated to English-are given in Appendix 1.) Each picture appeared twice in each language version: once with a negative, once with an affirmative target sentence. (For examples, see Fig. 1.) Items were pseudorandomized. There were never more than four consecutive negative or affirmative sentences.

### Scoring

All sentences constructed by the participants were written down and scored for polarity-related accuracy. The absence of the negative marker in target negative sentences and the presence of the negative marker in target affirmative sentences were scored as errors. Instances of incorrect ordering of the negative marker relative to the verb in the negative polarity condition (e.g., *Die Großmutter nicht zerschmettert die Untertasse.* ‘The grandmother not smashed the saucer (lit.)’) were also scored as errors. Cases of constituent negation (e.g., *La ragazza rifà non il letto*. ‘The girl makes not the bed (lit.)’) were scored as incorrect (and fell under the error type *incorrect ordering of the negative marker relative to the verb*) only if they were not consistent with the context provided by the picture.

### Data Analysis

Results were analyzed both at the individual and at group levels. We used Fisher’s exact tests for count data at the individual level and fitted generalized linear mixed-effects models to the datasets to analyze the data at the group level. Mixed models were fitted using the lme4 package (Bates et al., 2014). Since accuracy was coded as a dichotomous variable (correct or incorrect response at single-trial level), generalized mixed-effect models with logit transformation were fitted to the datasets (Jaeger, 2008). In both languages, Polarity had two levels: negative (i.e. negative sentences) and positive (i.e. affirmative sentences). We fitted models varying in complexity (e.g., models with and without random slopes) to identify those that provided the best fit for each dataset. We used the Akaike Information Criterion (AIC) (see Burnham & Anderson, 2004) for model selection. The best-fitting model for the German and Italian datasets included Group (PWAs, controls) and Polarity (negatives, affirmatives) as fixed effects, the interaction between Group and Polarity, Subjects and Items as random intercepts, and Polarity as by-Subject random slope. We also fitted models separately to the two datasets of PWAs (i.e. dataset of German-speaking PWAs and dataset of Italian-speaking PWAs). The best-fitting model for these datasets included Polarity (negatives, affirmatives) as fixed effect, Subjects and Items as random intercepts, and Polarity as by-Subject random slope. Lastly, to investigate the effect of verbal WM capacity and education on task performance, we merged the German and Italian datasets and fitted three models. The first model included verbal WM capacity/span and (years of formal) Education as fixed effects, and Subjects and Items as random intercepts. The second model included the interaction between verbal WM capacity/span and Polarity and the interaction between Education and Polarity as fixed effects, and Subjects and Items as random intercepts. The third model included all the terms included in the second model plus Polarity as by-Subject random slope. Model selection based on AIC showed that the third model was better than the second one.

## Results

**Fig. 2:**
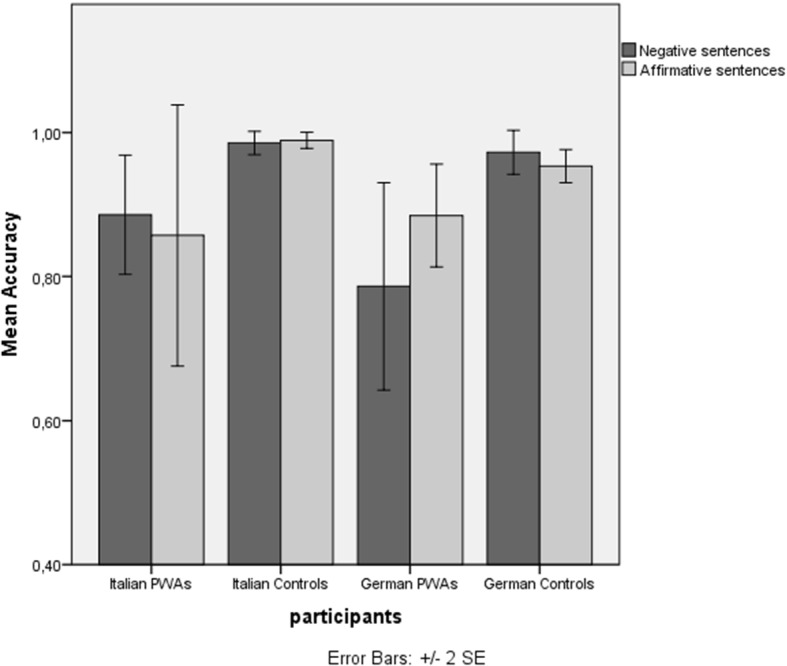
% correct performance on the production of negative and affirmative sentences

The results of the two outperforming models fitted to the datasets of the German-speaking and Italian-speaking participants are reported in Tables 4 and 5, respectively. Both language groups of PWAs fared worse than the corresponding control groups, the difference being significant for the Italian-speaking participants and marginally significant for the German-speaking participants (see also Fig. 2). Since the results of the best model fitted to the German dataset yielded a marginally significant interaction between Polarity and Group, we also fitted generalized mixed-effects models to the dataset of the German-speaking PWAs to find out whether these participants performed comparably on the production of negative and affirmative sentences. Indeed, this is what the results of the best model fitted to this dataset revealed (see Table 6). Lack of dissociation between negative and affirmative sentences was also observed in the group of Italian-speaking PWAs. This is shown both in Table 5, which shows that there is no main effect of Polarity and no significant interaction between Polarity and Group in the Italian dataset, and in Table 7, which presents the results of the best model fitted to the dataset of the Italian-speaking PWAs. Table 8 reports the output of the models fitted to the unified dataset (in which we merged the data of all participants) to explore whether verbal WM capacity and/or education affected task performance and whether either variable significantly interacted with the production of negative and affirmative sentences. As shown in Table 8, although neither variable showed a significant interaction, there was a significant main effect of verbal WM capacity on task performance; the greater the participants’ verbal WM capacity, the better their performance on the sentence anagram task.

**Table 4:** Generalized mixed-effects model on accuracy fitted to the dataset of the group of German-speaking PWAs and its matched control group (Model 1-German)

	Estimate	SE	z value	Pr( >|z|)
(Intercept)	2.570	0.430	5.974	*p* < 0.001*
Polarity = Negative	− 0.539	0.690	− 0.782	*p* = 0.434
Group = Control	1.074	0.558	1.925	*p* = 0.054
Polarity = Negative:Group = Control	1.962	1.031	1.903	*p* = 0.057

The model included the additive effect of Polarity and Group, the interaction between the two, subjects and items as random intercepts, and Polarity as by-subject random slope

The symbol * indicates significant effects

**Table 5:** Generalized mixed-effects model on accuracy fitted to the dataset of the group of Italian-speaking PWAs and its matched control group (Model 1-Italian)

	Estimate	SE	z value	Pr( >|z|)
(Intercept)	2.8857	0.7495	3.850	*p* < 0.001*
Polarity = Negative	− 0.1249	0.7466	− 0.167	*p* = 0.867
Group = Control	2.9322	1.0887	2.693	*p* < 0.01*
Polarity = Negative:Group = Control	− 0.5499	1.1247	− 0.489	*p* = 0.625

The model included the additive effect of Polarity and Group, the interaction between the two, subjects and items as random intercepts, and Polarity as by-subject random slope

The symbol * indicates significant effects

**Table 6:** Generalized mixed-effects model on accuracy fitted to the dataset of the group of German-speaking PWAs (Model 2-German)

	Estimate	SE	z value	Pr( >|z|)
(Intercept)	2.528	0.475	5.321	*p* < 0.001*
Polarity = Negative	− 0.610	0.547	− 1.115	*p* = 0.265

The model included Polarity as fixed effect, subjects and items as random intercepts, and Polarity as by-subject random slope

The symbol * indicates significant effects

**Table 7:** Generalized mixed-effects model on accuracy fitted to the dataset of the group of Italian-speaking PWAs (Model 2-Italian)

	Estimate	SE	z value	Pr( >|z|)
(Intercept)	2.791	0.781	3.576	*p* < 0.001*
Polarity = Negative	− 0.265	0.721	− 0.367	*p* = 0.714

The model included Polarity as fixed effect, subjects and items as random intercepts, and Polarity as by-subject random slope

The symbol * indicates significant effects

**Table 8:** Generalized mixed-effects models on accuracy fitted to the unified dataset (all participants collapsed)

	Estimate	SE	z value	Pr( >|z|)
Model 3				
(Intercept)	1.330	1.111	1.198	*p* = 0.231
Verbal WM capacity	0.523	0.136	3.857	*p* < 0.001*
Education	0.028	0.078	0.360	*p* = 0.719
Model 4				
(Intercept)	1.919	1.312	1.462	*p* = 0.144
Verbal WM capacity	0.516	0.163	3.164	*p* < 0.01*
Polarity = Negative	− 0.684	1.888	− 0.362	*p* = 0.717
Education	− 0.003	0.092	− 0.034	*p* = 0.973
Verbal WM capacity: Polarity = Negative	0.108	0.236	0.457	*p* = 0.648
Education: Polarity = Negative	0.044	0.133	0.331	*p* = 0.741

Model 3 included the additive effect of verbal WM capacity/span and (years of formal) Education, and subjects and items as random intercepts. Model 4 included the interaction terms verbal WM capacity/span x Polarity and Education x Polarity as fixed effects, subjects and items as random intercepts, and Polarity as by-subject random intercept

The symbol * indicates significant effects

The individual results of the German-speaking and the Italian-speaking participants are presented in Tables 9 and 10, respectively. The lack of dissociation between negative and affirmative sentences at the group level is also reflected in the individual results, as only two out of nine German-speaking PWAs (P2 and P6) and one out of seven Italian-speaking PWAs (P2) exhibited a dissociation between the two conditions. It should be noted, however, that, while German-speaking P2 and P6 patterned together as they both performed significantly better on affirmative than on negative sentences, Italian-speaking P2 exhibited the opposite pattern. Thus, German-speaking P2 and P6 on the one hand, and Italian-speaking P2 on the other hand, made up a double dissociation. We will turn to this double dissociation in the Discussion. Finally, all 25 healthy control participants performed comparably on negative and affirmative sentences.

**Table 9:** Performance of German-speaking PWAs (P) and healthy controls (C): Count data (correct performance), percent accuracy, standard deviation, and statistical comparisons using Fisher’s exact test for count data

	Negatives (*N* = 26)	Affirmatives (*N* = 26)	Total (*N* = 52)	Negatives vs. Affirmatives
P1	25 (96%)	24 (92%)	49 (94%)	*p* = 1
P2	13 (50%)	24 (92%)	37 (71%)	*p* < 0.01*
P3	26 (100%)	26 (100%)	52 (100%)	*p* = 1
P4	25 (96%)	26 (100%)	51 (98%)	*p* = 1
P5	19 (73%)	18 (69%)	37 (71%)	*p* = 1
P6	10 (38%)	21 (81%)	31 (60%)	*p* < 0.01*
P7	23 (88%)	23 (88%)	46 (88%)	*p* = 1
P8	20 (77%)	25 (96%)	45 (87%)	*p* = 0.099
P9	23 (88%)	20 (77%)	43 (83%)	*p* = 0.465
C1	25 (96%)	24 (92%)	49 (94%)	*p* = 1
C2	26 (100%)	24 (92%)	50 (96%)	*p* = 0.490
C3	26 (100%)	26 (100%)	52 (100%)	*p* = 1
C4	26 (100%)	25 (96%)	51 (98%)	*p* = 1
C5	26 (100%)	25 (96%)	51 (98%)	*p* = 1
C6	26 (100%)	25 (96%)	51 (98%)	*p* = 1
C7	25 (96%)	24 (92%)	49 (94%)	*p* = 1
C8	21 (81%)	25 (96%)	46 (88%)	*p* = 0.191
C9	26 (100%)	26 (100%)	52 (100%)	*p* = 1
C10	26 (100%)	26 (100%)	52 (100%)	*p* = 1
C11	26 (100%)	22 (85%)	48 (92%)	*p* = 0.110
C12	26 (100%)	24 (92%)	50 (96%)	*p* = 0.490
C13	26 (100%)	25 (96%)	51 (98%)	*p* = 1
C14	23 (88%)	26 (100%)	49 (94%)	*p* = 0.235

The symbol * indicates significant effects

**Table 10:** Performance of Italian-speaking PWAs (P) and healthy controls (C): Count data (correct performance), percent accuracy, standard deviation, and statistical comparisons using Fisher’s exact test for count data

	Negatives (*N* = 25)	Affirmatives (*N* = 25)	Total (*N* = 50)	Negatives vs. Affirmatives
P1	23 (92%)	25 (100%)	48 (96%)	*p* = 0.490
P2	19 (76%)	8 (32%)	27 (54%)	*p* < 0.01*
P3	25 (100%)	23 (92%)	48 (96%)	*p* = 0.490
P4	18 (72%)	22 (88%)	40 (80%)	*p* = 0.289
P5	23 (92%)	24 (96%)	47 (94%)	*p* = 1
P6	22 (88%)	24 (96%)	46 (92%)	*p* = 0.609
P7	25 (100%)	24 (96%)	49 (98%)	*p* = 1
C1	23 (92%)	24 (96%)	47 (94%)	*p* = 1
C2	25 (96%)	24 (96%)	49 (98%)	*p* = 1
C3	25 (96%)	25 (96%)	50 (100%)	*p* = 1
C4	25 (100%)	25 (100%)	50 (100%)	*p* = 1
C5	25 (100%)	24 (96%)	49 (98%)	*p* = 1
C6	24 (96%)	25 (100%)	49 (98%)	*p* = 1
C7	25 (100%)	25 (100%)	50 (100%)	*p* = 1
C8	25 (100%)	25 (100%)	50 (100%)	*p* = 1
C9	25 (100%)	25 (100%)	50 (100%)	*p* = 1
C10	24 (96%)	25 (100%)	49 (98%)	*p* = 1
C11	25 (100%)	25 (100%)	50 (100%)	*p* = 1

The symbol * indicates significant effects

### Error Analysis

Table 11 presents the distribution of the polarity-related errors. Errors are classified into three types: *incorrect absence of the negative marker* (in the negative polarity condition), *incorrect presence of the negative marker* (in the positive polarity condition), and *incorrect ordering of the negative marker relative to the verb* (in the negative polarity condition). The first two error types are the most frequent in all four groups (see Table 11).

**Table 11:** Polarity-related error distribution

	German PWAs	Italian PWAs	German controls	Italian controls
Incorrect absence of negative marker in the negative polarity condition	39.5% (30/76)	39.0% (16/41)	33.3% (9/27)	57.1% (4/7)
Incorrect ordering of negative marker relative to the verb in the negative polarity condition	25.0% (19/76)	2.4% (1/41)	3.7% (1/27)	0% (0/7)
Incorrect presence of negative marker in the positive polarity condition	35.5% (27/76)	58.5% (24/41)	63.0% (17/27)	42.9% (3/7)

The German-speaking PWAs and healthy controls performed 392/468 (83.8%) and 701/728 (96.3%) correct (in both the negative and positive polarity conditions), respectively. Thus, they made 76 and 27 polarity-related errors in total (in both conditions), respectively. The Italian-speaking PWAs and healthy controls performed 309/350 (88.3%) and 543/550 (98.7%) correct, respectively. Therefore, they made 41 and 7 polarity-related errors in total, respectively

It should also be noted that in all cases of *incorrect ordering of the negative marker relative to the verb* that occurred in the group of German-speaking PWAs, the negative marker *nicht* preceded the verb. In the group of German-speaking PWAs, there were also 23 instances of a Verb Second (V2) violation in the negative polarity condition. In V2 languages (e.g., German, Dutch) the verb of the matrix (i.e., unembedded) clause has to appear in second position (e.g., *Ich schlafe nicht* ‘I sleep not (lit.)’ “I am not sleeping”) (Koster, 1975). This grammatical rule is known as V2. This rule does not apply to embedded clauses in German, which is verb final for such clauses (e.g., *dass ich nicht schlafe* ‘that I not sleep (lit.)’ “that I am not sleeping”)*.* Of these 23 errors, there were 19 responses in which the V2 violation co-occurred with incorrect ordering of the negative marker relative to the verb, and one in which the V2 violation co-occurred with incorrect absence of the negative marker. Thus, all the *incorrect ordering of the negative marker* errors produced by the German-speaking PWAs co-occurred with V2 violation errors.

Of the 19 responses in which the V2 violation co-occurred with incorrect ordering of the negative marker relative to the verb, 13 were SNegVO sentences (e.g., *Die Frauen nicht waschen die Decke* ‘The women not wash the blanket (lit.)’), 5 were SONegV sentences (*Die Frau die Teller nicht zerbricht* ‘The woman the plates not breaks (lit.)’) and 1 was an SNegOV sentence (*Der Gärtner nicht die Blumen pflückt* ‘The gardener not the flowers picks (lit.)’).

In the same group, there were nine instances of V2 violation in the positive polarity condition, three of which co-occurred with incorrect presence of the negative marker. Thus, of the German-speaking PWAs’ 207 responses in the positive polarity condition that were scored as correct for polarity, there were only six cases (2.9%) of a V2 violation (e.g., *Die Frau die Teller säubert*. ‘The woman the plates cleans (lit.)’, *Der Mann den Braten wendet.* ‘The man the spit turns (lit.)’, *Die Oma die Fernbedienung hält*. ‘The grandma the remote control holds (lit.)’). Furthermore, in the group of German-speaking healthy controls, there were also three instances of a V2 violation in the negative polarity condition. Of these, one co-occurred with incorrect ordering of the negative marker relative to the verb (*Die Großmutter nicht zerschmettert die Untertasse.* ‘The grandmother not smashed the saucer (lit.)’). There were no instances of a V2 violation in the positive polarity condition.

Finally, in the group of the Italian-speaking PWAs, the only *incorrect ordering of the negative marker relative to the verb* error was a case of constituent negation where the negative marker *non* followed the verb and preceded the object (i.e. *La ragazza rifà non il letto*. ‘The girl makes not the bed (lit.)’). However, this case of constituent negation was inappropriate to the context provided by the picture.

## Discussion

This study investigated the ability of German-speaking and Italian-speaking PWAs to construct negative and affirmative sentences using a sentence anagram task modeled after Rispens et al. (2001). Given the different syntactic properties of the German and Italian negative markers examined (*nicht* and *non*, respectively), most of the accounts of (morpho)syntactic impairment in non-fluent aphasia discussed here-namely, Bastiaanse et al.’s (2002) account, the revised version of the IFIH (Fyndanis, Arfani, et al., 2018), Hagiwara’s (1995) account, and the TPH (Friedmann & Grodzinsky, 1997)-make different predictions for the performance of the two PWA groups in the production of sentential negation, as a function of the language and of the linguistic analysis of sentential negation one adopts (predictions are summarized in Table 1). Results failed to show significant difference between negative and affirmative sentences in either language group of PWAs.

Since the German negative marker *nicht* does not block or interfere with verb movement, lack of dissociation between negative and affirmative sentences for German-speaking PWAs supports Bastiaanse et al.’s (2002) view that sentential negation is compromised only if it blocks verb movement or otherwise interferes with it. On Belletti’s (1990, 1994) proposal that the Italian preverbal negative marker *non* interferes with verb movement, lack of dissociation between negative and affirmative sentences for Italian-speaking PWAs contradicts Bastiaanse et al.’s (2002) hypothesis, however. Results for Italian are consistent with Bastiaanse et al. (2002) only if one adopts Zanuttini’s (1997, 2001) view that *non* does not interfere with verb movement.

Results from the present study are not consistent with the original version of the IFIH (Fyndanis et al., 2012; Nanousi et al., 2006; Varlokosta et al., 2006), as this hypothesis would expect both PWA groups to perform significantly worse on negative sentences than on affirmative sentences. They are in line, though, with the predictions of the revised IFIH (Fyndanis, Arfani, et al., 2018) if one also adopts Zanuttini’s (2001) proposal that the Italian marker *non* does not have an affixal status (and thus is not a bound morpheme). Nevertheless, following Belletti’s (1990, 1994) analysis that the negative marker *non* is a clitic (and thus a bound morpheme), results for Italian are at odds with the revised IFIH (Fyndanis, Arfani, et al., 2018).

Finally, the results for both German and Italian are consistent with the TPH (Friedmann & Grodzinsky, 1997) and Hagiwara’s (1995) account only if one adopts Belletti’s (1990, 1994) view that NegP is located below TP in the syntactic hierarchy of Italian. Under Zanuttini’s (2001) proposal that NegP is located above TP in the Italian syntactic tree, only the German results are consistent with the TPH (Friedmann & Grodzinsky, 1997) and Hagiwara’s (1995) account.

Thus, the present results can be accounted for by all the hypotheses discussed except for the original version of the IFIH (Fyndanis et al., 2012). However, the Italian results can only be accounted for by these hypotheses if the “appropriate” syntactic analysis of sentential negation for Italian is adopted. They can be accounted for by Bastiaanse et al.’s (2002) hypothesis and by the revised IFIH (Fyndanis, Arfani, et al., 2018) only under Zanuttini’s (2001) analysis of sentential negation in Italian. The same results can be accounted for by the TPH (Friedmann & Grodzinsky, 1997) and Hagiwara’s (1995) hypothesis only under Belletti’s (1990, 1994) syntactic analysis of the Italian sentential negation. This study, therefore, illustrates the importance of syntactic theory and of choosing between competing theoretical approaches when it comes to accounting for patterns of (morpho)syntactic production in PWAs and to testing relevant hypotheses (for more details regarding the relationship between linguistic theory and aphasia research, see Garraffa & Fyndanis, 2020).

It should also be noted that the output of the data analysis at the group level aligns with the individual results as only 3 of the 16 PWAs reported here (German-speaking P2 and P6, and Italian-speaking P2) exhibited a dissociation between negative and affirmative sentences. Nevertheless, the performance of these three PWAs instantiated an unexpected double dissociation. While German-speaking P2 and P6 performed worse on negative than on affirmative sentences, Italian-speaking P2 displayed the opposite pattern (see Tables 9 and 10). There is no clear-cut explanation for the unexpected direction of the dissociation in Italian-speaking P2. However, since this participant was 101 months post-onset of his stroke, it is possible that resorting, perhaps inadvertently, to the more demanding marked polarity value resulted from a response strategy adopted during the task.

As expected, all 25 healthy control participants performed comparably on negative and affirmative sentences, which is consistent with healthy participants’ ceiling performance on similar anagram tasks in Rispens et al. (2001), Bastiaanse et al. (2002), and Fyndanis et al. (2006). Although negation has been found to be cognitively demanding (e.g., Albu et al., 2021; Kaup & Dudschig, 2020, and references therein), the comparably high performance of healthy participants in the current study is not surprising as neurotypical individuals have normal processing resources. Furthermore, the outcome measure of the anagram task used here was accuracy performance, which is not as sensitive as reaction times to the difference in processing demands between negative and affirmative sentences. We will return to this issue later in the discussion.

The significant main effect of verbal WM capacity on accuracy performance is consistent with earlier findings showing that verbal WM is involved in verb-related morphosyntactic processing (e.g., Fyndanis, Arcara, et al., 2018; Kok et al., 2007). Absence of a significant main effect of education on task performance could be accounted for by assuming that education is a proxy for long-term WM for language (Caplan & Waters, 2013), and that this procedural memory system is not involved in metalinguistic language tasks like sentence anagram (see “Limitations and Future Research” section). In contrast, controlled memory systems such as verbal WM are recruited in metalinguistic tasks.

### Discussion Related to the Error Analysis

The error analysis shows that, in all groups of PWAs and healthy participants, the most frequent error types relate to *incorrect absence of the negative marker* and *incorrect presence of the negative marker*. These two error types are connected to both conditions of the sentence anagram task employed here (i.e. *negative polarity* and *positive polarity*). Since the same error pattern was exhibited by PWAs and healthy controls, this possibly reflects task effects (e.g., misinterpretation of pictures) rather than a tendency of PWAs to omit free-standing grammatical morphemes such as negative markers (as one would expect based on the literature on non-fluent aphasia). In other words, most errors produced by the PWAs do not seem to result from a selective impairment of sentential negation. Lastly, the fact that 25% of the errors produced by the German-speaking PWAs consisted of incorrect ordering of the negative marker relative to the verb does not necessarily mean that these participants had a selective impairment in the syntax of sentential negation, since in all cases of incorrect ordering of the negative marker there was also a violation of the V2 rule.

The fact that the German-speaking PWAs made V2 violation errors in only 2.9% (6/207) of the affirmative sentences that they constructed in the positive polarity condition—which were scored as correct for polarity—suggests that their V2 violation errors in the negative sentences they constructed in the negative polarity condition are due to the interaction between the syntax of sentential negation and the V2 rule in German. In other words, it may be that the German-speaking PWAs were mainly impaired in the syntax of sentential negation, which also resulted in V2 violation errors in the negative polarity condition. An alternative explanation is that, in the negative polarity condition, the combined demands of sentential negation and the V2 rule taxed the processing system of the German-speaking PWAs, resulting in a general word order impairment affecting different aspects of grammar and leading to multiple syntactic errors. In fact, in most cases it is hard to tease apart syntactic errors related to sentential negation and syntactic errors related to the violation of the V2 rule. This alternative explanation is also supported by the observation that the Italian-speaking PWAs only made one incorrect ordering of the negative marker error—note that the Italian language does not have the V2 rule. Finally, it should be noted that most responses in which a V2 violation co-occurred with incorrect ordering of the negative marker relative to the verb could not have been treated as embedded clauses. This is so because, of the 19 responses in which a V2 violation error co-occurred with incorrect ordering of the negative marker relative to the verb, only 5 were SONegV sentences, that is, sentences featuring the canonical word order for embedded clauses in German.

### Discussion Related to Processing Demands of Negation and Sentence Anagram Task

As mentioned in the Introduction, negative sentences have consistently been found to pose more demands on the processing system than affirmative sentences, as negation prolongs processing times (e.g., for a recent review, see Kaup & Dudschig, 2020). This is especially true when negative sentences are presented out of context or with insufficient context (e.g., Albu et al., 2021; for recent reviews, see Dudschig et al., 2021a, and Kaup & Dudschig, 2020), as was the case for the sentence anagram task with pictures used in Rispens et al. (2001), Bastiaanse et al. (2002), and here (for details, see the “Introduction” section). Nevertheless, as shown in this and earlier studies (e.g., Bastiaanse et al., 2002), the asymmetry in processing costs is not always reflected in participants’ accuracy performance on negative vs. affirmative sentences. Probably this is so because accuracy measures are not always sufficiently sensitive. However, in Bastiaanse et al.’s (2002) and Rispens et al.’s (2001) cross-linguistic studies on non-fluent aphasia, in which accuracy performance was the dependent variable, dissociations were reported in some languages but not in others. This suggests that language-specific syntactic properties of negation pose indeed *extra* demands on the processing system of PWAs, which may at times be captured even by accuracy performance, a measure which is less sensitive than reaction times.

### Limitations and Future Research

This study is not free of limitations. The main limitation is that the conclusions are based on a sentence anagram task, which could be considered as being metalinguistic. It could be argued that this task poses demands beyond sentence planning and that, therefore, results reflect task effects rather than (or, in addition to) PWAs’ ability to produce negative and affirmative sentences. The same objection could be leveled against not only sentence anagrams but also other frequently used tasks, such as sentence completion and picture description (e.g., Hofstede & Kolk, 1994; Nerantzini et al., 2020). However, despite the possible task effects, control groups outperformed PWAs, which shows that the sentence anagram task distinguishes neurotypical speakers from participants with brain damage. Moreover, when versions of the same task were administered to Dutch-speaking, English-speaking, Norwegian-speaking, Spanish-speaking, and Greek-speaking PWAs (Bastiaanse et al., 2002; Fyndanis et al., 2006; Rispens et al., 2001), different patterns and levels of impaired performance emerged depending on the language. Specifically, in the studies above, the production of sentential negation was found impaired in English, Spanish and Greek, but not in Dutch and Norwegian. Given that the same anagram task was used in all five languages, the reported cross-linguistic discrepancies in the impairment/preservation of sentential negation cannot be reduced to task effects. Hence, we argue that, despite possible task effects, the anagram task can measure an impairment of sentential negation. Lastly, since Bastiaanse et al.’s (2002) negation-specific hypothesis was based on sentence anagram tasks, we decided to use a similar task to ensure comparability with the cross-linguistic results reported in the original paper.

Another limitation of the study relates to the fact that accuracy performance was selected as the dependent variable (aka outcome measure), which presumably is not sensitive enough to the increased processing demands associated with negation (e.g., Albu et al., 2021; Dudschig et al., 2021a; Kaup & Dudschig, 2020). It is possible that only when the increased processing demands linked to negation are coupled with *additional* processing demands associated with *language-specific* (morpho)syntactic properties of negation does the computational asymmetry between negative and affirmative sentences translate into significantly worse accuracy performance on negative than on affirmative sentences. To clarify this point, additional tasks (e.g., tasks eliciting verbal responses, differing in processing demands, and using more sensitive measures/dependent variables than accuracy-e.g., reactions times) should be used in future research to tease apart the effects of task and those of negation, and thus to draw firmer conclusions regarding the ability of PWAs to produce sentential negation. Moreover, since cognitive inhibitory mechanisms are involved in negation processing (Beltrán et al., 2018, 2019; 2021; Dudschig et al., 2021b), future research should investigate the role of verbal WM (which affected task performance in the current study) and cognitive inhibitory mechanisms in the production of sentential negation.

A second limitation results from the relatively small sample sizes, which do not allow one to generalize findings to the general population of German-speaking and Italian-speaking PWAs. Larger numbers of PWAs should be tested in future research to increase the statistical power of the studies.

Lastly, future research should aim at differentiating between the more promising accounts discussed above. To this end, sentential negation in aphasia should be tested in more languages that differ in the critical syntactic properties of the negative markers. Furthermore, negation should be tested in a broader context, i.e. together with other verb-related morphosyntactic/functional categories (e.g., tense, mood, aspect, complementizer), as the predictions made by the TPH (Friedmann & Grodzinsky, 1997), Hagiwara (1995), and the revised IFIH (Fyndanis, Arfani, et al., 2018) cover the whole syntactic hierarchy.

### Concluding Remarks

This study contributes to the cross-linguistic database regarding the production of sentential negation in non-fluent aphasia by reporting data from German-speaking and Italian-speaking PWAs. Even though PWAs fared worse than neurotypical participants in both German and Italian, neither language group was found to be impaired in the production of sentential negation relative to the production of affirmative sentences, consistent with various accounts of (morpho)syntactic impairment in non-fluent aphasia, such as Bastiaanse et al.’s (2002) account, Friedmann and Grodzinsky’s (1997) TPH, Hagiwara’s (1995) account, and Fyndanis, Arfani, et al.’s (2018) revised IFIH. The study also highlights the importance of syntactic theory in accounting for patterns of (morpho)syntactic production exhibited by PWAs. The ability of PWAs to produce sentential negation should be explored in more languages, and in larger samples. Future studies on the topic should also be linguistically informed as linguistic theory can provide powerful descriptive and explanatory tools that enable a deeper understanding of patterns of performance exhibited by PWAs and cross-linguistic differences.

## Appendix 1: Sentence Anagram Task Administration Instructions

(translation from Italian to English-the German instructions were similar to the Italian ones) *NB: Instructions for the experimenter/examiner are in italics. Please familiarize yourself with the PowerPoint presentation and read through all the instructions in advance to prepare yourself for administering the task*.

I will show you some pictures, one at a time, along with a set of cards. Each card has words written on it. Your task is to try to put the cards in the right order to form a correct sentence. All sets of cards contain the word “not”. However, the use of the card with “not” is optional, since it depends on the picture.

For example, I will show you this picture *(we show the first picture of the PPT presentation, i.e. the first picture of the “practice items”)* and I will give you these cards *(we present the participant with the first set of cards; we show the cards one by one and read aloud clearly each word that is written on them):* [is carving] [the turkey] [not] [the lady]. Well, what can you see in this picture? Is the lady curving the turkey? *(We shall wait a few seconds for the participant’s answer*). NO, as we can see, the lady is placing/putting potatoes in the baking tin with the turkey. In this case, we need to use the card with the word [not] to form the correct sentence “The lady is not carving the turkey” ([the lady] [not] [is carving] [the turkey] *lit.*).

Another example: I will show you this picture *(we present the second picture from the* *“practice items”**)* and I will give you these cards *(we give the participant the second set of cards in the same order they are placed in the card holder; we show the cards one by one and read aloud clearly each word on them)*: [is connecting] [not] [the electrician] [the cables]. The picture depicts an electrician who is connecting cables. So, to form the correct sentence we should leave aside the card with “not” and form the sentence “The electrician is connecting the cables” ([the electrician] [is connecting] [the cables]).

Is that clear? Let’s see some other examples. *The experimenter presents/continues with the remaining practice items until (s)he makes sure that the participant has understood the task. Should the participant still have doubts about the task after having completed all the practice items, the experimenter explains again the task, running the practice trials again from the beginning. Once the experimenter makes sure that the participant has understood the task, (s)he should proceed to the experimental part, which starts with the slide “Experimental items”. The experimenter writes down the sentences formed by the participant for each picture*. *Note: In the ppt presentation of the sentence anagram task, below the pictures (in “Notes”) you can see both the target sentence and the bracketed sentence fragments/constituents in a pseudorandomised order. Each bracketed sentence fragment represents a card. All participants will be given all these cards in the same pseudorandomized order*.
